# New Treatment Perspectives in Autism Spectrum Disorders

**DOI:** 10.3389/fped.2015.00022

**Published:** 2015-03-18

**Authors:** Roberto Canitano, Yuri Bozzi

**Affiliations:** ^1^Division of Child Neuropsychiatry, University Hospital of Siena, Siena, Italy; ^2^Laboratory of Molecular Neuropathology, Centre for Integrative Biology (CIBIO), University of Trento, Trento, Italy; ^3^CNR Neuroscience Institute, Pisa, Italy

**Keywords:** autism spectrum disorders, autism, autism spectrum disorders and treatment, diagnostic markers, pharmacology

In the past 10 years, research on autism spectrum disorders (ASD) has made a considerable progress, leading to the identification of a number of genes and signaling pathways associated to ASD pathogenesis. Nevertheless, our understanding of the complexity of these disorders remains elusive, and effective treatments are still lacking (1). In this e-book, we present 16 articles addressing several different aspects of both clinical and basic research on ASD. A particular emphasis is put on the efforts that are currently made to identify reliable diagnostic markers and novel therapeutic strategies, as well as on the progress of ongoing clinical trials.

Though ASD are recognized as cross-cultural disorders, discrepancies in early diagnosis and interventions are present in western countries. Genetic testing procedures in Europe and USA are still discordant; these issues are addressed by Amiet and coworkers (2). New ASD treatments are emerging and deserve to be mentioned. A number of novel medications have been used off-label in various studies, including drugs approved for Alzheimer’s disease, as reviewed by Rossignol and Frye (3). Among these, it has to be outlined that in a large multisite controlled study memantine was not shown to improve core and associated symptoms in ASD and the open phase of the trial was in fact suspended. Similarly, cholinesterase inhibitors did not show substantial modifications in ASD core symptoms and their use is still not warranted. Drugs commonly used to treat mitochondrial diseases such as L-carnitine, complex B vitamins, antioxidants etc. have been found to improve ASD symptoms in some studies, but results are still conflicting and more research is needed. As a whole, the field is active and in progress but reports are discordant and do not yet allow us to draw firm conclusions regarding safety and efficacy. Further, research is needed to define subgroups of children with ASD in which these treatments may be most effective (4). ASD children treated with a ketogenic diet (KGD) showed decreased seizure frequencies and improved learning abilities and social skills, as proposed by Napoli and coworkers (5); however, replications of this investigation are urgently awaited to have of a clearer picture of KGD role in ASD. Excitation and inhibition (E/I) imbalance in ASD has been demonstrated in preclinical models, and targeted treatments directed either to reduce excessive glutamatergic transmission or increase inhibition through stimulation of GABAergic signaling have been introduced with promising preliminary results. The implication of oxytocin in social development and affiliative behaviors has been ascertained and findings from clinical trials in children with ASD showed encouraging results especially in social cognition. Stimulation of excitatory synapses and neuronal density has been achieved with insulin-like growth factor 1 (IGF-1) administration and it has been positively tested in two single gene disorders associated with ASD, Rett syndrome, and Phelan-McDermid syndrome. These preliminary clinical trials point to additional research in larger samples. Notably, modifications of neural pathways of ASD have been observed after behavioral developmental interventions through the evaluation by functional neuroimaging and electroencephalography, providing evidence of a dynamic neural substrate susceptible of functional changes. This new conceptualization paves the way to a modern treatment approach to this group of disorders once thought as hard wired and not amenable of changing, as discussed in the papers by Pini and colleagues (6) and Canitano (7).

The role of melatonin in the establishment of circadian rhythms and the synchronization of peripheral oscillators is probably linked to the synchrony of motor, emotional, and social rhythms that are altered in ASD. Potential therapeutic benefits of melatonin in the recovery of circadian rhythms have been demonstrated in a growing number of studies in ASD. Developmental behavioral interventions that emphasize synchrony (in some cases combined with melatonin) seem to provide substantial improvement in ASD, as reviewed by Tordjman (8) and reported by Fulton (9). Research on outcome of interventions is currently an active field of investigation, though data available do not allow to answer the question of “what works for whom” in ASD. This is critical to delineate the guidelines for behavioral interventions, as reviewed by Vivanti and colleagues (10). Among the bothersome ASD symptoms that need to be addressed, auditory hypersensitivities may be tackled by means of novel techniques such as the listening protocol projects, as proposed by Porges (11). Poor reciprocal social communication is a hallmark characteristic of ASD and is under intense investigation for treatment; Corbett and colleagues report a novel theatre intervention that combines interaction of trained peers with patients to facilitate the performance-based theatrical treatment (12).

The last part of the book is dedicated to basic studies highlighting the importance of investigating mouse models to unravel the molecular mechanisms underlying ASD pathogenesis. Two review articles, respectively by Cellot and Cherubini (13) and Giovedí and coworkers (14), address the role of GABAergic neurotransmission and synaptic vesicle proteins in the pathogenesis of ASD, strengthening the notion of ASD as a “synaptic neuropathology”. Finally, three original research articles investigate novel pathological aspects in three different ASD mouse models. Provenzano and colleagues show growth hormone and IGF-1 signaling deficits in Engrailed-2 mouse mutants (15); Gigliucci et al. demonstrate oxytocin signaling alterations in mice lacking opioid receptors (16), and Michetti and coworkers identify a series of pathological features (including defects in glutamatergic neurotransmission) in reeler mice (17). Taken together, these mouse studies confirm the importance of IGF-1, oxytocin, and glutamate signaling in the pathogenesis of ASD, as extensively described in several clinical studies presented in this e-book.

## Conflict of Interest Statement

The authors declare that the research was conducted in the absence of any commercial or financial relationships that could be construed as a potential conflict of interest.
